# Impact of Freeze Storage on the Estimation of Phenotypic Antimicrobial Resistance Prevalence in *Escherichia coli* Collected from Faecal Samples from Healthy Humans and Chickens

**DOI:** 10.3390/antibiotics11111643

**Published:** 2022-11-17

**Authors:** Bach Tuan Kiet, Nguyen Thi Nhung, Nguyen Thi Phuong Yen, Doan Hoang Phu, Nguyen Thi Thuy Dung, Lam Kim Yen, Ho Thi Viet Thu, Juan J. Carrique-Mas

**Affiliations:** 1 Sub-Department of Animal Health and Production, Dong Thap 81000, Vietnam; 2 Oxford University Clinical Research Unit, Ho Chi Minh City 70000, Vietnam; 3 Faculty of Animal Sciences and Veterinary Medicine, Nong Lam University, Ho Chi Minh City 70000, Vietnam; 4 Faculty of Agriculture and Aquaculture, Dong Thap Community College, Dong Thap 81000, Vietnam; 5 Department of Agriculture, Can Tho University, Can Tho 94000, Vietnam; 6 Food and Agriculture Organization of the United Nations, Hanoi 10000, Vietnam; 7 Centre for Tropical Medicine, Nuffield Department of Clinical Medicine, Oxford University, Oxford OX3 7FZ, UK

**Keywords:** sample storage, antimicrobial resistance, *Escherichia coli*, humans, chickens

## Abstract

Many studies on phenotypic antimicrobial resistance (AMR) of bacteria from healthy populations are conducted on freeze-stored samples. However, the impact of this practice on phenotypic AMR is not known. We investigated the prevalence of phenotypic AMR in *Escherichia coli* from chicken (*n* = 10) and human (*n* = 11) faecal samples collected from healthy subjects, subject to freeze storage (−20 °C and −80 °C) for 1, 2, 3, and 6 months. We compared counts of *E. coli* and prevalence of phenotypic resistance against five antimicrobials commonly used in chicken farming (ciprofloxacin, enrofloxacin, doxycycline, gentamicin, and florfenicol) with samples processed within 24 h of collection. Prevalence of phenotypic AMR was estimated by performing differential counts on agar media with and without antimicrobials. At −20 °C, there was a considerable reduction in *E. coli* counts over time, and this reduction was greater for human samples (−0.630 log_10_ units per 100 days) compared with chicken samples (−0.178 log_10_ units per 100 days). For most antimicrobials, AMR prevalence estimates decreased in freeze-stored samples both in humans and chickens over time. Based on these results, we conclude that results on the prevalence of phenotypic AMR on samples from freeze-stored samples are unreliable, and only fresh samples should be used in such studies.

## 1. Introduction

Antimicrobial resistance (AMR) has been declared by the World Health Organization (WHO) as one of the top 10 threats affecting humanity [1]. Commensal *Escherichia coli* are commonly used as indicator organisms to measure levels of phenotypic AMR in the gut of healthy human and animal populations [2,3]. These organisms are intrinsically susceptible to most clinically relevant antimicrobial agents, and have the capacity to accumulate AMR-encoding genes through horizontal gene transfer [4]. Furthermore, *E. coli* can be an important vehicle for the dissemination of antimicrobial resistant genes (ARG) [5].

Prevalence levels of resistance in these organisms often correlate with levels of antimicrobial use (AMU) in animals [6] and in humans [7]. Studies on AMR are often carried out using commensal *E. coli* strains from faecal samples from healthy animals and humans. Such studies involve the recovery of isolates [8,9,10] and further antimicrobial susceptibility testing. Prevalence of resistance is often calculated by comparing numbers of *E. coli* colonies recovered from samples plated onto agar media with and without the target antimicrobial [10].

On many occasions, laboratory testing is not possible immediately after sample collection, and faecal samples or their eluates are often stored in freezing conditions to be conveniently tested later. This is often carried out in the context of metagenomic analyses (i.e., analysis of genomes contained in faecal samples), but in some cases also for the investigation of phenotypic AMR (i.e., prevalence of resistance). Some studies suggest that freeze storage of samples may not substantially affect the microbiome profile over a short or long time period [11,12,13]. The extent to which freeze storage may affect phenotypic AMR on *E. coli* isolates is, however, not known. It has been shown that many AMR traits entail fitness costs to bacteria, especially those encoded by mutations [14]. Because of this, we hypothesise that storage conditions may result in reduced survival of the more resistant strains, and therefore, culture work on freeze-stored samples may provide unreliable estimates of phenotypic resistance.

We compared estimates of phenotypic resistance in randomly selected *E. coli* from human and chicken flock samples stored in glycerol at two different temperature conditions (−20 °C and −80 °C), and compared these results with those obtained from faecal material immediately processed. An understanding of the impact of freeze storing conditions on AMR is relevant when planning studies aiming at investigating the prevalence of phenotypic AMR in *E. coli*.

## 2. Results

### 2.1. Counts of E. coli Colonies in Fresh and Stored Samples

**Figure 1:**
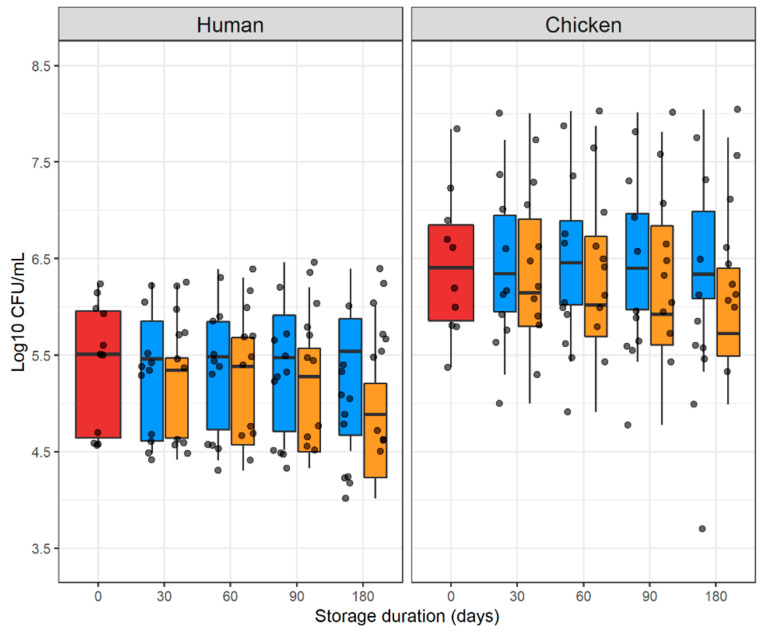
Boxplots displaying median and 25% and 75% quartile of counts of *E. coli* on ECC plates within 24 h after collection, as well as following storage at −20 °C and −80 °C after 1, 2, 3, and 6 months. Red, fresh sample; blue, −80 °C; orange, −20 °C.

Baseline counts (i.e., fresh samples) were 321.5 (IQR 44.4–907.5) × 10^3^ and 2848 (IQR 730.5–7165) × 10^3^ cfu/mL in human and chicken samples, respectively (Figure 1). The *E. coli* counts of each sample are presented in Table S1.

Overall, storage of samples at −80 °C over time resulted in slightly increased *E. coli* counts in both human and chicken samples. However, at −20 °C, there was a considerable reduction in counts over time, which was quantitatively greater for human samples (−0.630 per 100 days) compared with chicken samples (−0.178 per 100 days, Table 1).

### 2.2. Prevalence of Resistance over Time in Faecal Samples

**Figure 2:**
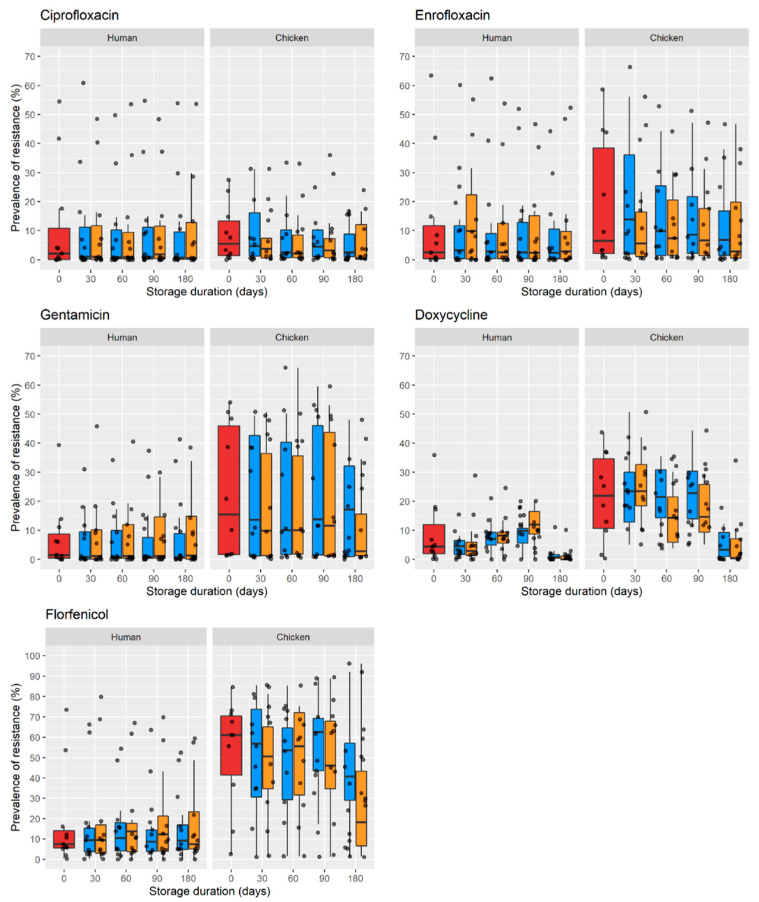
Prevalence of phenotypic AMR among *E. coli* from human and chicken fresh and freeze-stored faecal samples after 30, 60, 120, and 180 days. Red, fresh sample; blue, −80 °C; orange, −20 °C.

The prevalence of phenotypic AMR among *E. coli* in human and chicken samples is shown in Figure 2. Predictions from the models are shown in Table 2. The parameters and coefficients of these models are provided in Table S2. The estimated prevalence of resistance decreased over time for most antimicrobials tested, except for gentamicin in human samples, which was predicted to increase from 21% to 51% after 300 days at −20 °C. For chicken samples, the prevalence of resistance reduced more markedly in samples stored at −80 °C compared with −20 °C. For human samples, the opposite was the case, except for florfenicol, whose prevalence declined faster at −20 °C.

## 3. Discussion

Storage of faecal samples for later isolation and subsequent phenotypic AMR investigation is relatively common practice in many microbiological laboratories. Our results suggest that storage at −80 °C resulted in good preservation of *E. coli* bacteria, consistent with a previous study [15]. We hypothesise that the observed increases in *E. coli* counts in samples stored at −80 °C may be linked to the differential mortality of other bacterial species in the sample, thus providing *E. coli* with a competitive advantage. It has been shown that freezing faecal samples may change the bacterial community structure [16], although some studies have only found marginal changes based on 16S rRNA analyses [11]. *Escherichia coli* have been generally described as relatively resilient organisms under different environmental conditions [17]. We suggest investigating this using spiked samples, mixing *E. coli* with a number of other enteric bacteria at known concentrations and measuring the changes over time in freezing conditions.

For most antimicrobials, AMR prevalence estimates decreased in freeze-stored samples both in humans and chickens over time. These findings are consistent with a recent study on *E. coli* recovered from freeze-stored (−80 °C) faecal samples, reporting measurable decreases in tetracycline and amoxicillin resistance after 6–12 months compared with fresh samples [18]. The single exception in our study was gentamicin resistance in human samples, which surprisingly increased its prevalence over time at −20 °C. A plausible explanation is that under these conditions, *E. coli* isolates not harbouring gentamicin-resistance-encoding genes had comparatively less mortality. A previous study on pig faecal samples showed a general decline in ARG abundance after freeze storage (−20 °C and −80 °C) compared with samples immediately processed. The exception was for macrolide- and β-lactam-encoding ARGs [19]. However, that study was conducted on two samples only.

Freeze storage of chicken samples at −80 °C resulted in less marked reductions in the prevalence of resistance than storage at −20 °C. However, for human samples, resistance declined more markedly when stored at −80 °C for four of the five antimicrobials tested. This phenomenon, alongside the increases in *E. coli* counts at −80 °C, is a puzzling finding that requires further investigation. Potentially, this could be investigated by carrying out controlled studies with mixtures containing known quantities of gentamicin-resistant *E. coli* bacteria alongside other enteric human bacteria under freezing conditions over time.

Although we believe that the sample size is adequate and includes a representative number of unrelated chicken flocks and human subjects (farmers), there is still a small possibility that these results may not be comparable with samples containing very different enteric flora and antimicrobial resistance patterns. We therefore recommend to confirm this by conducting studies on a range of animal species and variable resistance traits.

## 4. Materials and Methods

### 4.1. Study Design

We estimated the prevalence of phenotypic AMR among *E. coli* from chicken (n = 10) and human (n = 11) faecal samples stored under −20 °C and −80 °C conditions, after 1, 2, 3, and 6 months, and compared these results with samples processed within 24 h of collection. Prevalence of phenotypic AMR was estimated by performing differential counts on agar with and without antimicrobials.

### 4.2. Sample Collection

Ten different meat chicken flocks sampled at ages 1–4 weeks located in the Mekong Delta province of Dong Thap (Vietnam) were investigated. All flocks were single-age; each consisting of 200–500 birds housed in confinement. In each chicken pen/house, sterile paper liners were placed near drinkers and feeders to collect fresh droppings. After a minimum of 10 droppings had been deposited, liners were swabbed using sterile gauze. Each collected gauze was placed in a universal jar. Samples were collected by staff affiliated to the Sub-Department of Animal Health and Production of Dong Thap (SDAHP-DT). Rectal swabs were collected from chicken farmers by Center for Disease Control of Dong Thap (CDC-DT) staff. Each swab was placed immediately into a vial containing 5 mL of BHI + glycerol 20% and was transferred (at 4 °C) to the laboratory within 24 h of collection. In the laboratory, human rectal swabs were vortexed thoroughly to release and suspend the faecal matter in the liquid medium. Subsequently, 5 g of each chicken faeces were suspended in 45 mL of BHI + glycerol 20%. For each sample, two sets of five aliquots (0.5 mL of sample/aliquot) were stored at −20 °C and −80 °C, respectively.

### 4.3. Estimation of the Prevalence of Resistant E. coli

A volume of 100 µL of sample matrix was diluted to 1:100 (human samples) and 1:1000 (chicken samples) in saline solution. ECC agar (CHROMagar, Paris, France) plates with and without antimicrobials were inoculated with 50 µL of sample matrix. The following five antimicrobials were investigated: gentamicin, 8 mg/L (aminoglycoside class); ciprofloxacin, 2 mg/L; enrofloxacin, 1 mg/L (fluoroquinolone class); doxycycline, 8 mg/L (tetracycline class); and florfenicol, 8 mg/L (amphenicol class). The quality of the plates was controlled using susceptible reference (*E. coli* ATCC 25922) as well as in-house *E. coli* strains resistant to each of the antimicrobials tested.

All plates were incubated at 37 °C for a period of 18–20 h. The total number of suspected *E. coli* (blue colonies) were counted from the ECC agar plates with and without antimicrobials. In ECC agar, *E. coli* form either a distinct blue colony or white colony in appearance with a small blue centre. We used MALDI-TOF MS (MALDI Biotyper 3.1, version 3.1.65, MBT library 5627 mps, Bruker Daltonics, Billerica, MA, USA) to confirm the species identity of over 200 colonies.

The prevalence of resistance was calculated by dividing the counts of *E. coli* on both types of plate. We performed these analyses on fresh samples (i.e., within 24 h after sample collection), and on samples stored at −20 °C and −80 °C after 1, 2, 3, and 6 months. All experiments were conducted in duplicate.

### 4.4. Data Analyses

We built Poisson regression random effects models for counts of chicken and human *E. coli* isolates. The sample ID was included as a random effect. We modelled the interaction of time and freezing conditions (−80 °C vs. −20 °C) as variables of interest. For studies on counts in antimicrobial plates relative to total counts (i.e., in non-antimicrobial plates), we included the number of colonies in the non-selective plates (log) as offset in the model. All models were built using the lme4 package in R.

## 5. Conclusions

We conclusively demonstrate here the importance of sample storage in the investigation of phenotypic resistance in *E. coli* from faecal samples from humans and animals. Based on these results, we do not recommend carrying out studies on frozen samples, given that this may lead to false results, generally resulting in an underestimation of the prevalence of phenotypic AMR.

## Figures and Tables

**Table 1:** Predictions derived from a Poisson model of the effects of storage duration and temperature on counts of *E. coli* colonies in fresh and freeze-stored human and chicken samples.

Sample	Days	Human ^1^	Chicken ^2^
Fresh	0	235,626	2,520,581
−20 °C	30	194,989	2,389,513
	100	125,367	2,109,581
	300	35,490	1,477,704
−80 °C	30	244,850	2,707,943
	100	267,801	3,201,084
	300	345,933	5,162,854

^1^ Intercept: 12.37 (SE ± 0.17); ^1^ Coeff. for −20 °C (100 days): −0.630; ^1^ Coeff. for −80 °C (100 days): 0.128; ^2^ Intercept: 14.74 (SE ± 0.04); ^2^ Coeff. for −20 °C (100 days): −0.178; ^2^ Coeff. for −20 °C (100 days): 0.239. (All *p*-values < 0.001).

**Table 2:** Predictions derived from a Poisson model of the effects of storage duration and temperature on the change in prevalence (%) of phenotypic AMR in human and chicken samples.

Host	Antimicrobial	Model-Predicted Prevalence of Resistance (%)						
		Fresh Sample	Storage at −20 °C			Storage at −80 °C		
			30 d	100 d	300 d	30 d	100 d	300 d
Human	Ciprofloxacin	0.054	0.053	0.051	0.046	0.053	0.049	0.040
	Enrofloxacin	0.08	0.081	0.078	0.069	0.077	0.065	0.041
	Doxycycline	4.46	4.28	3.89	2.97	3.89	2.82	1.12
	Gentamicin	0.21	0.23	0.29	0.51	0.21	0.19	0.15
	Florfenicol	7.65	7.25	6.39	4.45	7.47	7.04	5.97
Chicken	Ciprofloxacin	3.88	2.92	1.50	0.22	3.45	2.64	1.22
	Enrofloxacin	6.99	5.96	4.11	1.42	6.65	5.90	4.19
	Doxycycline	20.60	17.10	11.08	3.21	18.60	14.66	7.43
	Gentamicin	10.03	8.32	5.38	1.55	9.27	7.72	4.57
	Florfenicol	41.48	35.35	24.34	8.38	39.37	34.85	24.61

## Data Availability

Not applicable.

## Supplementary Materials

The following supporting information can be downloaded at: https://www.mdpi.com/article/10.3390/antibiotics11111643/s1. Table S1: CFU/mL of *E. coli* in presence/ absence of antimicrobials. Table S2: The parameters and coefficients of models investigate the effects of storage duration and temperature on the change of phenotypic AMR.
